# Mucosal Melanoma of the Head and Neck: A 45-Year Experience of a Tertiary Cancer Center

**DOI:** 10.3390/cancers18081304

**Published:** 2026-04-20

**Authors:** Stefano Cavalieri, Benedetta Lombardi Stocchetti, Andrea Spagnoletti, Francesco Barretta, Andrea Anichini, Patrizia Boracchi, Gabrina Tragni, Lorenza Di Guardo, Alice Indini, Barbara Valeri, Roberto Bianchi, Sarah Colombo, Nicola Alessandro Iacovelli, Marzia Franceschini, Michele Del Vecchio, Marco Guzzo

**Affiliations:** 1 Head and Neck Medical Oncology Department, Fondazione IRCCS Istituto Nazionale dei Tumori, 20133 Milan, Italy; 2 Department of Oncology and Hemato-Oncology, University of Milan, 20122 Milan, Italy; 3 Melanoma Medical Oncology Unit, Medical Oncology Department, Fondazione IRCCS Istituto Nazionale dei Tumori, 20133 Milan, Italy; 4 Department of Biostatistics for Clinical Research, Fondazione IRCCS Istituto Nazionale dei Tumori, 20133 Milan, Italy; 5 Department of Experimental Oncology, Fondazione IRCCS Istituto Nazionale dei Tumori, 20133 Milan, Italy; 6 Medical Statistics Unit, Department of Biomedical and Clinical Sciences, University of Milan, 20122 Milan, Italy; 7 Pathology Department, Fondazione IRCCS Istituto Nazionale dei Tumori, 20133 Milan, Italy; 8 Maxillo-Facial Surgery Unit, Otolaryngology and Head and Neck Surgery Department, Fondazione IRCCS Istituto Nazionale dei Tumori, 20133 Milan, Italy; 9 Radiotherapy Department, Fondazione IRCCS Istituto Nazionale dei Tumori, 20133 Milan, Italy

**Keywords:** mucosal melanoma, prognostic factors, head and neck melanoma

## Abstract

Background/Objectives. Head and neck mucosal melanoma (HNMM) is a rare, aggressive malignancy with poor outcomes and limited evidence to guide prognostication and treatment. A detailed assessment of long-term survival and prognostic factors is needed to inform clinical management and staging. This work aimed to describe outcomes and prognostic factors in HNMM patients treated over 45 years. Methods. This was a retrospective observational cohort study of consecutive patients treated at a tertiary referral center in Italy from 1975 to 2020. Random-forest-based screening informed covariate selection for Cox models. Endpoints were overall survival (OS), disease-free survival (DFS), and post-recurrence DFS (prDFS). Associations with clinical and pathological variables were evaluated using Kaplan–Meier estimates, log-rank tests, and multivariable Cox regression. Results. Among 112 patients (median follow-up, 121.1 months), 3-/5-year OS was 42.8%/28.0%, DFS 20.5%/13.2%, and 1-/3-year prDFS 36.7%/10.9%. Ulceration was associated with worse OS (HR 2.12; 95% CI 1.05–4.26) and DFS (HR 2.23; 95% CI 1.16–4.28). Male sex showed a trend toward poorer OS and DFS. Regional lymph-node treatment correlated strongly with OS and prDFS (overall *p* < 0.001), with neck dissection indicating unfavorable risk (OS HR 5.22; 95% CI 2.39–11.40). Conclusions. HNMM remains a high-mortality disease with frequent recurrence. Ulceration and nodal involvement were key adverse prognostic factors, while surgery was associated with improved survival. The findings support incorporating ulceration into future staging and highlight the potential for durable control through salvage surgery. Further investigation of treatment intensification, biomarkers, and multimodal strategies is warranted.

## Simple Summary

Mucosal melanoma of the head and neck is a rare and aggressive cancer with limited evidence to guide treatment decisions and predict patient outcomes. Because of its rarity, most available data come from small or heterogeneous series, making it difficult to identify reliable prognostic factors. In this study, we analyzed patients treated over a 45-year period at a single specialized cancer center to better understand long-term outcomes and factors associated with survival. We found that certain pathological features, such as tumor ulceration, and clinical factors, including lymph node involvement, are strongly linked to worse prognosis, while surgery—especially when complete resection is achievable—remains crucial for disease control. These findings may help improve risk stratification, inform future staging systems, and guide treatment strategies, ultimately contributing to more personalized care for this challenging disease.

## 1. Introduction

Head and neck mucosal melanoma (HNMM) is a rare and aggressive malignancy arising from melanocytes located in the mucosa of the upper respiratory and digestive tracts. It most frequently involves the nasal cavity, paranasal sinuses, and oral cavity, whereas the nasopharynx, oropharynx, larynx, middle ear, and upper esophagus are less commonly affected. HNMM accounts for less than 1% of all melanomas, approximately 4% of all sinonasal tumors, and 0.26% of all oral malignancies [1,1,2,3,4].

Unlike cutaneous melanomas (CMs), mucosal melanomas are characterized by a more aggressive biological behavior, often present at an advanced stage, and carry a significantly poorer prognosis. The etiology remains largely unknown. HNMM primarily affects older adults, with a peak incidence between the sixth and eighth decades of life, and no consistent predilection by sex or race has been demonstrated [5,6].

Outcomes are generally poor, with 5-year overall survival (OS) rarely exceeding 30% and disease-free survival (DFS) frequently below 10%. The most common causes of treatment failure are local recurrence and distant metastases [7]. Some literature data suggest that prognosis may be influenced by the primary site, with worse outcomes reported for paranasal melanomas [8,9]. Poor survival is further exacerbated by late onset and non-specific symptoms, delayed diagnosis, and the technical difficulty of achieving a complete surgical resection [10].

A tumor–node–metastasis (TNM) staging system specific to mucosal melanomas of the head and neck was first introduced in the AJCC/UICC 7th edition (2009) [11], acknowledging the unique biology and clinical behavior of these tumors. The recently released 9th edition (2025) [12] reaffirms these distinct features, maintaining a separate classification for HNMM and underlining its poor prognosis compared to cutaneous forms.

Immunotherapy with immune checkpoint inhibitors (ICIs) and targeted therapies have dramatically improved outcomes of advanced CM patients. However, supporting a comparable survival benefit in HNMM is hard to define due to the rarity of this malignancy [13]. HNMMs have a lower tumor mutational burden and a distinct genomic profile, usually lacking BRAF V600 mutations and more commonly harboring alterations in KIT, NRAS, NF1, and SF3B1 [14]. These features limit the availability of effective targeted therapies (BRAF inhibitors + MEK inhibitors for BRAF mutated cancers, MEK inhibitors for NRAS ones, etc.). and highlight the need for site-specific prognostic models and treatment strategies.

Currently, surgery remains the cornerstone of treatment for locoregionally advanced HNMM, with or without adjuvant radiotherapy (RT). While adjuvant radiation improves locoregional control (probability of locoregional recurrence after surgery 29.9% vs. 55.6% with or without RT, respectively) [15,16,17], its impact on overall survival remains controversial, and no randomized data are available so far. Definitive RT is sometimes offered to inoperable patients. A Japanese study reported a series of 31 HNMM patients treated with definitive surgery (21 without surgery). In case of incomplete resection, RT did not improve either local control or survival [18]. However, no prospective direct comparison between surgery and definitive RT is available. Given the historical perception of mucosal melanoma as radioresistant, particle therapy—including carbon and proton therapy—has gained increasing attention and shown promising results in selected cases [19,20]. In particular, an Italian study on 40 HNMM patients treated with carbon ion RT reported a 2-year overall survival of 58.6%, with an improvement in terms of local and distant control against literature data [19].

In this context, we conducted a large-scale, retrospective analysis of patients with primary HNMM treated at a single tertiary cancer center in Italy over a 45-year period. The present work is a long-term update of a previous preliminary report published more than 30 years ago [21]. Our aims were to characterize the long-term natural history of HNMM, assess survival outcomes, and identify clinical and pathological prognostic factors. Leveraging real-world data and statistical modeling, including random forest and multivariable Cox regression, we investigated the applicability of traditional prognostic markers used in CM and sought to identify potential novel predictors for mucosal disease. Although CM and mucosal melanoma differ in terms of etiology and genomic landscape, several histopathological features—such as ulceration and lymph node involvement—reflect fundamental biological processes, including tumor proliferation, hypoxia, angiogenesis, and tumor–host interaction, which are not site-specific. On this basis, we hypothesized that selected CM-derived prognostic markers may retain clinical relevance in HNMM while also exploring potential disease-specific predictors.

## 2. Materials and Methods

This article reports the retrospective analysis of the institutional case series of patients accessing the Maxillo-facial surgery Unit at the Fondazione IRCCS Istituto Nazionale dei Tumori (Milan, Italy) from 1975 to 2020.

This observational study was approved by the Institutional Ethical Committee (Comitato Etico Fondazione IRCCS Istituto Nazionale dei Tumori di Milano) on 28 April 2022 (local study ID INT 74/22). This study was conducted in compliance with the Declaration of Helsinki, as per Good Clinical Practice, and in the framework of Italian law and the European General Data Protection Regulation.

Medical charts (both paper-based and electronic health records) were reviewed to collect data about clinical presentation at diagnosis, treatments, pathologic features, date and site of disease relapse, and oncologic outcomes.

Patients were stratified based on treatment date before or after 2010. This cutoff was selected to approximate a major transition in the management of HNMM, during which several relevant advances were progressively introduced into clinical practice, including intensity-modulated radiotherapy (IMRT), the availability of ICIs, increased access to particle therapy, and the implementation of a dedicated TNM staging system. In addition, this time point reflects an institutional shift in treatment strategy, with a relative decrease in surgical approaches at our institution and increased use of non-surgical modalities, particularly particle therapy such as carbon ion radiotherapy. Although these changes were not introduced simultaneously nor uniformly adopted, 2010 was used as a pragmatic inflection point to distinguish between historical and more contemporary treatment eras. This variable was included for exploratory purposes to account for potential temporal trends, rather than to infer causal effects.

### Statistical Methods

Demographic and clinical data were collected from medical records in a centralized anonymized database. Variables were selected based on (i) clinical relevance according to existing literature on melanoma and head and neck cancers, (ii) availability and completeness in the institutional database, and (iii) biological plausibility as potential prognostic factors (e.g., tumor burden, tumor microenvironment, and treatment-related variables).

Given the exploratory nature of this study, we adopted a data-driven approach using random-forest-based screening to reduce dimensionality and identify the most informative variables for subsequent multivariable modeling.

Retrieved data included sex (male, female), age (years), primary tumor site (nasal cavity, oral cavity, other, unknown), neck lymph node status/site (cranial neck, medium neck, cN0 [no clinical lymph node involvement]), symptoms at diagnosis (bleeding, mass, nasal obstruction, other, none), clinical regional lymph node involvement (yes, no), year of treatment for primary tumor (before 2010, after 2010 included, unknown), primary treatment including surgery (yes, no), treatment of regional lymph nodes (neck dissection, none/biopsy, cN0 [no clinical lymph node involvement]), radiotherapy (yes, no), site of recurrence (no recurrence, local, regional, distant), status (alive, death), year of treatment for recurrence (before 2010, 2010 or later, no recurrence), treatment for recurrence (no recurrence, including surgery, not including surgery), Breslow thickness (mm), histologic type (mucosal lentiginous, nodular, other, not specified), previous pigmentation (yes, no), pigmentation (yes, no), cytologic features (epithelioid, other, not specified), infiltration (none, connective tissue, bone, other), ulceration (absent, present, not specified), regression (absent, present, not specified), tumor-infiltrating lymphocytes (TILs: absent, brisk, not brisk, not specified), mitoses (#/mm^2^), lymphovascular invasion (absent, present, not specified), vascular neogenesis (absent, present, not specified), necrosis (absent, present, not specified), perineural invasion (absent, present, not specified), satellitosis (absent, present, not specified), sclerosis (absent, present, not specified), lateral surgical margins (no surgery, negative, positive, not specified/not applicable), deep surgical margins (no surgery, negative, positive, not specified/not applicable), date of symptoms beginning, date of primary tumor treatment beginning, date of lymph node treatment beginning, date of radiotherapy beginning, and dates of first and second relapse.

The aims of this study were overall survival (OS), disease-free survival (DFS) and post-recurrence disease-free survival (prDFS) in HNMM patients. OS was defined as the time from the start of primary-tumor treatment to death from any cause. DFS was defined, among patients who were disease-free after primary treatment, as the time from the start of primary-tumor treatment to the first recurrence or death, whichever occurred first. prDFS was defined, among patients who experienced a recurrence after being disease-free following primary treatment, as the time from the diagnosis of recurrent disease to the next recurrence/progression or death, whichever occurred first. For all endpoints, patients who were alive and event-free according to the endpoint definition were censored at the date of last follow-up. OS, DFS, and prDFS curves were estimated using the Kaplan–Meier method and compared using the log-rank test to assess univariable association between the endpoint and a selected group of variables.

Multivariable Cox models were fitted to assess the influence of selected variable on each endpoint. The results are presented as hazard ratios (HRs) with 95% confidence intervals (CIs) and Wald test *p*-values.

Continuous variables were described by median and by first and third quartiles (referred to as IQR). Categorical variables were described as absolute and relative frequencies. The median follow-up was estimated with the reverse Kaplan–Meier method [22].

To select variables for inclusion in the association analysis and in the multivariable Cox models, three random forest procedures [23] (specific for survival framework) were used on available data to estimate the probability of OS (n = 112), DFS (n = 102), and prDFS (n = 90). Available data refer to patients with complete information on all variables. The variables were selected from those listed in Table 1.

Given the relatively small sample size for a data driven-approach, and the exploratory nature of this study, random forest was used as a screening tool to rank candidate covariates rather than as a predictive modeling approach. This strategy was intended to reduce dimensionality before regression modeling; however, variable-importance estimates in small datasets may be unstable and should be interpreted cautiously.

Variables were selected based on their relative importance according to a permutation test *p* value [24,25]. One thousand datasets were created by permuting the response variable with 1000 classification trees included in each permutation of the response variable. The null hypothesis means that, for each variable, the relative importance calculated in the real set of origin is no better than the relative importance calculated in each permutated dataset. Controlling for false positives due to multiple testing was done by adjusting the permutation test *p* values (indicating relative importance) using the Benjamini–Hochberg procedure [26]. All covariates retained at the screening step for at least one endpoint were subsequently evaluated for all endpoints in univariable analyses. In multivariable modeling, each endpoint was fitted including only the covariates selected for that specific endpoint at screening; in case of collinearity among candidate covariates, we retained the variable with the highest variable-importance measure.

On the selected variables, interactions between couples of variables were assessed by means of “effect importance measure” (EIM) in “interaction forest”. EIM quantifies the extent to which the joint effect of two variables contributes to outcome prediction beyond their individual effects, thus capturing potential interaction effects. Briefly, EIM is computed through a permutation-based approach that evaluates the change in model performance when the interaction structure between two variables is disrupted while preserving their marginal distributions. Larger EIM values indicate stronger interaction effects between variables. Given the exploratory nature of this analysis, the interaction results were interpreted cautiously. Analyses were performed using SAS Studio V04.00 (Cary, NC) and R V4.5.1 [27].

## 3. Results

### 3.1. Patient Characteristics

A total of 112 patients (56 women, 50%) were included in the analysis, with a median follow-up of 121.1 months (first and third quartiles: 97.1–162.6 months). Median age was 60 years (first and third quartiles: 52.5–70.0). Patient and treatments characteristics are described in Table 2, and pathologic characteristics of the primary disease are described in Table 3. The occurrence of regional lymph nodes preceded the diagnosis of primary melanoma in three cases (2.7%).

### 3.2. Variable Selection

Using random forest screening, variables were shortlisted for association testing if they were significant after multiplicity adjustment and/or showed a nominal *p* < 0.10. For OS, treatment of regional lymph nodes (adjusted *p* < 0.001) and primary tumor treatment (adjusted *p* = 0.042) were the only features remaining significant after adjustment; sex, RT, ulceration, and lymph-node site met the nominal *p* < 0.10 criterion (*p* = 0.048, 0.073, 0.084, and 0.066, respectively). For DFS, no variables survived multiplicity adjustment; ulceration (*p* = 0.018) and sex (*p* = 0.050) met the nominal criterion. For prDFS, treatment of regional lymph nodes remained significant after adjustment (adjusted *p* = 0.046), while sex met the nominal criterion (*p* = 0.082).

### 3.3. Oncologic Outcomes

**Figure 1:**
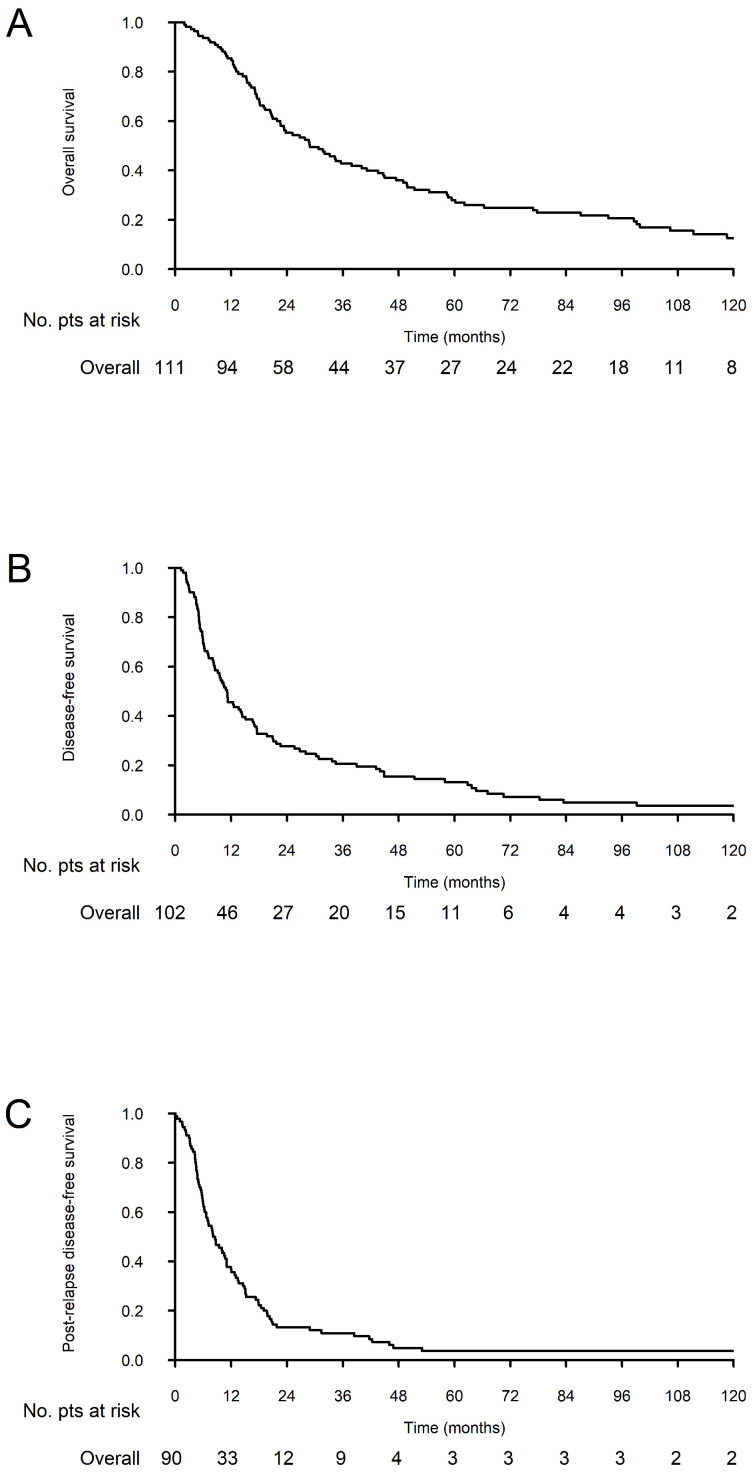
Overall (**A**), disease-free (**B**) and post-relapse disease-free (**C**) survivals.

In the overall cohort, 3- and 5-year OS were 42.8% (34.4–53.2%) and 28.0% (20.5–38.1%), while 3- and 5-year DFS were 20.5% (13.9–30.2%) and 13.2% (7.9–21.9%). One- and 3-year prDFS were 36.7% (28.0–48.1%) and 10.9% (6.0–19.8%) (Figure 1).

No significant differences were observed between outcomes according to the year of primary tumor treatment (Supplementary Figure S1).

### 3.4. Association Analysis

According to univariate analysis, primary tumor site was not associated with OS, DFS, or prDFS (Supplementary Figure S2; *p* = 0.968, *p* = 0.530, and *p* = 0.816, respectively). Three- and 5-year OS were 41.3% (30.6–55.8%) and 27.0% (17.7–41.3%) for nasal cavity, 41.9% (28.8–61.0%) and 28.0% (16.6–47.0%) for oral cavity, and 50.0% (25.0–100.0%) and 25.0% (7.5–83.0%) for other sites; corresponding DFS estimates were 21.8% (13.4–35.4%) and 10.2% (4.5–22.9%), 14.3% (6.3–32.2%) and 11.4% (4.6–28.8%), and 33.3% (10.8–100.0%) at both time points, respectively. One- and 3-year prDFS were 35.8% (25.0–51.4%) and 11.3% (5.3–24.1%), 37.5% (24.0–58.7%) and 9.4% (3.2–27.5%), and 40.0% (13.7–100.0%) and 20.0% (3.5–100.0%), respectively.

Survival curves stratified according to the selected clinical and pathologic parameters are reported in the Supplementary Materials (OS, Supplementary Figure S3; DFS, Supplementary Figure S4; prDFS, Supplementary Figure S5).

Sex was associated with OS (*p* = 0.014) and DFS (*p* = 0.015): 3- and 5-year OS were 52.1% (40.3–67.3%) and 36.2% (25.3–52.0%) in females vs. 33.4% (22.8–48.8%) and 19.6% (11.3–34.0%) in males; 3- and 5-year DFS were 24.0% (14.7–39.3%) and 19.6% (11.1–34.6%) vs. 17.2% (9.4–31.7%) and 6.5% (2.2–19.1%), respectively. prDFS did not differ by sex (*p* = 0.114), with 1- and 3-year prDFS of 47.7% (35.0–65.0%) and 10.9% (4.6–25.9%) in females vs. 26.1% (16.0–42.4%) and 10.9% (4.8–24.9%) in males.

Ulceration was associated with OS (*p* = 0.033) and DFS (*p* = 0.013): OS at 3 and 5 years was 69.2% (48.2–99.5%) and 61.5% (40.0–94.6%) in absence of ulceration vs. 41.3% (31.2–54.5%) and 23.1% (15.0–35.8%) in presence of ulceration; DFS at 3 and 5 years was 46.2% (25.7–83.0%) (both time points) vs. 17.6% (10.4–29.7%) and 7.2% (2.8–18.4%). prDFS did not differ by ulceration (*p* = 0.259): 1- and 3-year prDFS were 54.5% (31.8–93.6%) and 18.2% (5.2–63.7%) vs. 35.6% (25.2–50.2%) and 10.2% (4.8–21.7%), respectively.

Treatment of regional lymph nodes differed for OS (*p* < 0.001) and prDFS (*p* < 0.001), but not for DFS (*p* = 0.479). For OS, 3- and 5-year estimates were 20.8% (9.6–45.4%) and 8.3% (2.2–31.4%) after neck dissection, 66.4% (51.3–86.0%) and 50.4% (34.7–73.4%) with none/biopsy, and 39.6% (28.4–55.2%) and 24.5% (15.3–39.3%) in the cN0 group. For prDFS, 1- and 3-year estimates were 11.1% (3.0–41.0%) and 0.0% (no patient at risk) after neck dissection, 53.8% (37.7–76.9%) and 26.9% (14.3–50.7%) with none/biopsy, and 37.8% (26.0–55.0%) and 6.7% (2.2–19.9%) in cN0.

The site of involved neck lymph nodes differed for OS (*p* < 0.001) and prDFS (*p* < 0.001). Three- and 5-year OS were 63.9% (48.8–83.6%) and 48.5% (33.0–71.1%) for cranial neck, 22.7% (10.5–49.1%) and 9.1% (2.4–34.1%) for medium neck, and 39.6% (28.4–55.2%) and 24.5% (15.3–39.3%) for cN0; corresponding 1- and 3-year prDFS were 50.0% (34.5–72.4%) and 25.0% (13.2–47.5%), 13.3% (3.7–48.4%) and 0.0%, and 37.8% (26.0–55.0%) and 6.7% (2.2–19.9%), respectively.

Primary surgery was associated with OS (*p* < 0.001), with 3- and 5-year OS of 45.6% (36.8–56.6%) and 30.5% (22.6–41.3%) in the surgery group vs. 11.1% (1.8–70.5%) and 0.0% in patients without surgery; no differences were observed for DFS (*p* = 0.322) or prDFS (*p* = 0.914). Radiotherapy was associated with OS (*p* = 0.004): 3- and 5-year OS were 46.5% (37.1–58.2%) and 33.2% (24.5–44.9%) without radiotherapy vs. 26.5% (12.7–55.3%) and 5.3% (0.8–35.4%) with radiotherapy; DFS (*p* = 0.676) and prDFS (*p* = 0.109) did not differ by radiotherapy.

### 3.5. Multivariable Analyses

In the multivariable Cox models (Table 4), OS was lower in patients with ulceration present compared with ulceration absent (HR 2.12, 95% CI 1.05–4.26; *p* = 0.035). OS was worse in patients receiving neck dissection (HR 5.22, 95% CI 2.39–11.40) compared to subjects without lymph node involvement. Male sex showed borderline evidence of worse OS vs. female (HR 1.64, 95% CI 1.00–2.71; *p* = 0.051). Primary tumor treatment was close to conventional significance, with better OS for surgery vs. no surgery (HR 0.24, 95% CI 0.05–1.17; *p* = 0.077).

For DFS, ulceration present was associated with worse DFS vs. absent (HR 2.23, 95% CI 1.16–4.28; *p* = 0.016), and male sex was close to significance with worse DFS vs. female (HR 1.57, 95% CI 0.98–2.49; *p* = 0.058).

For prDFS, outcomes differed by treatment of regional lymph nodes (overall *p* < 0.001), with worse prDFS for neck dissection (HR 4.25, 95% CI 2.21–8.18) and cN0 vs. none/biopsy (HR 1.67, 95% CI 1.00–2.80). Male sex was also close to significance and indicated worse prDFS vs. female (HR 1.48, 95% CI 0.96–2.28; *p* = 0.079).

### 3.6. Long-Term Survivors

Eight patients (7%) were alive and disease-free after at least 10 years of follow-up. Median age at diagnosis was 54 years (IQR 13.5), and they were mostly women (5), with nasal cavity primary melanoma (4). They were all treated with surgery, without RT. Six recurred at loco-regional level (four local, two regional), and all of them underwent salvage surgery with negative margins before 2010. None of these long-term survivors received immunotherapy either in the curative or recurrent/metastatic setting.

Given the very limited number of long-term survivors, no formal statistical comparisons were performed. However, descriptively, most experienced locoregional recurrence and underwent successful salvage surgery with negative margins. These observations should be interpreted as hypothesis-generating rather than conclusive.

## 4. Discussion

To the best of the authors’ knowledge, this is the largest case series of HNMM managed at a single institution.

The main findings of this study can be summarized as follows: First, long-term outcomes of HNMM remain poor, with high rates of recurrence and limited survival. Second, among clinical and pathological variables, ulceration and regional lymph node involvement emerged as the most relevant adverse prognostic factors, while primary tumor site was not associated with survival. Third, treatment-related variables showed that surgery and salvage surgery were associated with improved outcomes, although these findings are likely influenced by selection bias. Finally, a small subset of patients achieved long-term disease control, often in the context of successful salvage surgery.

Our results showed that the clinical characteristics of the analyzed patients are in line with the available literature. Most (76%) HNMM patients experience disease recurrence, and patients with regional lymph node involvement have worse prognosis than those without, as expected from the available literature [28]. Regional recurrence was observed in 11% of cases. Given that the threshold for recommending elective neck dissection is usually set at 20%, these data confirm the absence of evidence to recommend a neck dissection in patients with clinically node-negative (cN0) HNMM [29,30]. Unlike cutaneous melanoma, sentinel lymph node biopsy has a limited and controversial role, with reported positivity rates and prognostic impact varying widely across small retrospective series, and without clear evidence supporting its routine use [30,31].

As far as pathologic features are concerned, our results showed that the majority of cases had no pigmentation (87.5%), and presented ulceration (84%) or infiltration (77%). Only a minority had TIL brisk (14%), lympho-vascular (13%) or perineural (7%) infiltration, sclerosis (11%), regression (4%) or satellitosis (1%). In cases undergoing surgery, a radical resection (R0) was obtained in 79% of cases.

In terms of oncologic outcomes, differently from other literature reports [8], primary HNMM site did not affect survival. This finding differs from some previous reports suggesting worse outcomes for paranasal sinus primaries. Possible explanations include the predominance of nasal and oral cavity tumors in our cohort, and potential differences in staging accuracy and treatment strategies over time. In addition, the long study period and heterogeneity in diagnostic work-up may have attenuated site-specific effects. In the analyzed patient cohort, favorable prognostic factors included clinical characteristics (women had more favorable prognosis than men), pathologic features (absence of ulceration), and treatments (patients undergoing curative-intent surgery had more favorable outcomes; neck dissection likely reflected cN+ disease).

Approximately half of patients bearing disease recurrence received salvage surgery. Although uncommon (7%), long-term survivors shared a pattern of favorable clinical and pathological features and benefitted from effective salvage surgery, suggesting that durable disease control is achievable even after recurrence when complete resection is attainable. This finding corroborates the need to also pursue salvage surgery, whenever feasible [32], after the failure of innovative treatments such as hadron therapy, notably carbon ion RT (CIRT) [33]. Interestingly, when comparing our surgical-based cohort with more recent published series of HNMM treated with CIRT in Italy from 2013 onwards, the oncologic outcomes appear broadly comparable. Given the classical view of HNMM as a radioresistant malignancy, this similarity is notable and may suggest that high-LET (linear energy transfer) radiotherapies such as CIRT could mitigate some degree of radio-resistance of mucosal melanoma, potentially through more complex DNA damage.

This parallels experience in other historically radioresistant tumors—for example, soft tissue sarcomas or adenoid cystic carcinoma—where heavy-ion or high-LET radiation has resulted in improved local control compared with conventional photon RT. If these data are confirmed, CIRT (or other forms of hadron therapy) could represent a valid alternative to surgery for selected HNMM patients, particularly those with unresectable disease, questionable radical surgery or high surgical risk.

However, caution is warranted. Patient selection, staging uncertainties, and lack of long-term follow-up in published CIRT series may bias the comparison. In the absence of randomized data, it remains unclear whether the similar outcomes reflect true equivalence or are the result of selection of more favorable patients for CIRT (e.g., smaller tumors, absence of gross nodal disease, or good performance status).

This cohort was largely treated in the pre-immunotherapy era, limiting the direct applicability of our findings to current clinical practice. In HNMM, responses to ICIs appear generally lower compared to cutaneous melanoma, likely reflecting a distinct tumor biology characterized by lower tumor mutational burden and different genomic alterations. Available evidence in mucosal melanoma suggests modest response rates to anti-PD1 monotherapy and improved, yet still suboptimal, outcomes with combination strategies. However, in the advanced setting, anti-PD1 agents provide significant benefit in MM [34,35]. Adjuvant immunotherapy remains standard in cutaneous occurrences despite limited evidence specific to MM. CheckMate 238 enrolled only 29 MM patients without subgroup analysis [36], and retrospective data showed high relapse rates despite PD-1 therapy [37]. Neoadjuvant immunotherapy, particularly in combination with antiangiogenic agents (e.g., toripalimab + axitinib [38] or pembrolizumab + lenvatinib [39]), has shown promising response rates and survival outcomes in MM and warrants further investigation in HNMM.

The poor responsiveness of MM to ICIs compared to CM is well documented: in pooled analyses, the median PFS was 5.9 months for MM with anti-PD1 + anti-CTLA4 combo, compared to 11.7 months in CM [40,41]. Targeted therapy options are limited but may include KIT inhibitors, notably imatinib, for KIT-mutated MM [42,43]. Emerging strategies such as anti-PD1 + VEGF blockade (e.g., toripalimab + axitinib [44]; atezolizumab + bevacizumab [45]) appear promising in MM and are aligned with its vascular-rich biology.

### 4.1. Study Limitations and Strengths

Prognostic factors in HNMM have been extensively investigated in prior studies [46,47,48,49]. Although the present findings confirm existing evidence, the contribution to the field consists of the large validation of the available knowledge.

This work is not exempt from further limitations. First, the long period of observation implies heterogeneity in cancer treatments and patient outcomes. Nevertheless, with the caveat of the limited numbers of recently treated patients, no major differences were observed when stratifying patients according to treatment year. Second, there is likelihood of treatment indication bias due to the retrospective nature of the analysis. For instance, therapeutic neck dissection was performed only in patients with clinically evident nodal disease (cN+) [30]. Interestingly, patients who underwent neck dissection had significantly worse overall survival compared to those who did not. However, this finding should not be interpreted as a detrimental effect of surgery per se. Rather, it likely reflects confounding by indication, as neck dissection was performed almost exclusively in patients with clinically evident nodal disease (cN+), which is inherently associated with poorer prognosis. Therefore, neck dissection should be considered a surrogate marker of higher disease burden rather than an independent adverse prognostic factor.

This interpretation aligns with the current literature and is consistent with the staging rationale incorporated into the AJCC/UICC TNM classification.

The interpretation of the effect of primary surgery also warrants caution. Only a small proportion of patients did not undergo surgery, resulting in an imbalanced comparison and wide confidence intervals in multivariable analyses. This limits the robustness of the estimated effect size. Moreover, treatment allocation was not random and likely reflected clinical selection, with non-surgical approaches preferentially adopted in patients with more advanced, unresectable, or medically unfit disease. Therefore, the observed association between surgery and improved survival should not be interpreted as causal but rather as potentially influenced by selection bias.

Moreover, the interpretation of adjuvant RT outcomes should be approached with caution. In this retrospective cohort, patients who received postoperative RT had worse survival compared to those who did not. However, this likely reflects indication bias, as RT was often reserved for cases with high-risk pathological features or incomplete resections. Thus, poorer outcomes may stem from the underlying disease severity rather than the treatment modality itself. Indeed, literature data show the opportunity to consider adjuvant RT even in the presence of negative surgical margins [16,50,51,52].

Another major limitation of this study is the absence of complete TNM staging information for a substantial proportion of patients, particularly those treated before 2010. This reflects the historical context of the cohort: a dedicated TNM classification for HNMM did not exist prior to the 7th AJCC/UICC edition, and older cases often lacked standardized radiologic or pathological documentation. As a result, retrospective retrieval of original imaging or full pathology reports was not feasible. This predates the widespread adoption of immune checkpoint inhibitors (ICIs), which have substantially altered the therapeutic landscape of melanoma. Consequently, the clinical relevance of these findings to current practice is limited, and their applicability to contemporary treatment paradigms remains uncertain. Nonetheless, while this limits the ability to perform stage-adjusted analyses, it also mirrors real-world clinical practice in the pre-2010 era, underscoring the challenges of studying rare malignancies across long observational periods. Finally, no molecular data are available.

Acknowledging the cited limitations, this work has several strengths. Apart from being the largest single-institution case series published to date, the extended observation period enabled a comprehensive characterization of the natural history of the disease. As far as treatments are concerned, the cohort was relatively homogeneous, being essentially a surgical case series. Importantly, the comprehensive pathologic data let uncover the details of prognostic factors which are often underreported while managing HNMM.

For instance, ulceration was associated with unfavorable prognosis. Literature data report that the prognostic impact of ulceration in HNMM remains controversial [53]. In this scenario, the presence of ulceration modifies stage in cutaneous melanoma but not in HNMM. Given our results, we may speculate the community might consider incorporating ulceration in the next staging systems. To this end, validation in larger and multicenter cohorts is needed.

### 4.2. Future Directions

Future research should aim to validate the prognostic role of key factors identified in this study, particularly ulceration, in independent and preferably multicenter cohorts. Given the rarity of HNMM, collaborative efforts and registry-based studies will be essential to achieve adequate statistical power. Integration of molecular and genomic data is also needed to refine risk stratification and better understand the biological mechanisms underlying tumor aggressiveness.

In addition, prospective studies are warranted to evaluate the role of modern therapeutic strategies, including immunotherapy and particle therapy, in HNMM patients. Finally, any proposed modification in staging systems—such as the inclusion of ulceration—should be supported by consistent evidence across multiple datasets before being implemented in clinical practice.

## 5. Conclusions

Our results confirm that HNMM patients still have an unfavorable prognosis; so, treatment intensification is needed. In light of recent literature, potential strategies may be hypothesized, although evidence specific to HNMM remains limited. Among them, short-course neoadjuvant combination immunotherapy (anti-PD1 plus anti-CTLA4) before locoregional treatment may represent an investigational approach, but this hypothesis requires prospective validation in HNMM [54].

When available, hadron therapy, notably CIRT, may represent a promising option in unresectable cases; however, current evidence remains preliminary and is mainly based on retrospective and single-institution experiences. The potential role of adjuvant immunotherapy after RT or CIRT, particularly in patients with high-risk pathological features such as ulceration, remains to be defined and should be explored in prospective clinical studies.

Finally, the identification of reliable biomarkers is crucial to improve treatment selection in HNMM. Candidate biomarkers of interest may include PD-L1 expression, tumor mutational burden, KIT alterations, and features of the tumor microenvironment. Their integration into clinical decision-making may help identify patients more likely to benefit from immunotherapy, both in early-stage and advanced disease, thereby maximizing treatment benefit while minimizing unnecessary toxicity.

## Figures and Tables

**Table 1:** Variables included in the random forest selection procedures.

**Patient factors** Patient’s age at diagnosis Sex Symptoms at diagnosis
**Primary tumor factors** Ulceration Lymph node site Vascular neogenesis Previous pigmentation Pigmentation Necrosis Sclerosis Cytologic features Breslow Tumor-infiltrating lymphocytes Infiltration Lymphovascular invasion Primary tumor site Mitoses
**Treatments** Primary tumor treatment Treatment of regional lymph nodes Radiotherapy in the curative setting Year of primary tumor treatment

**Table 2:** Patients and treatments characteristics.

	N (%)
**Sex**	
Female	56 (50)
Male	56 (50)
**Age (years)**	
Median (first and third quartiles)	60 (52.5–70.0)
**Primary tumor site**	
Nasal cavity	63 (56)
Oral cavity	40 (36)
Other	8 (7)
Unknown	1 (1)
**Symptoms at diagnosis**	
Bleeding	34 (30)
Mass	41 (36)
Nasal obstruction	22 (20)
Other	12 (11)
None	3 (3)
**Clinical regional lymph node involvement**	
Yes	59 (53)
No	53 (47)
**Year of treatment for primary tumor**	
Before 2010	102 (92)
After 2010 included	9 (8)
Unknown	1 (1)
**Primary treatment including surgery**	
Yes	103 (92)
No	9 (8)
**Treatment of regional lymph nodes**	
Neck dissection	25 (22)
None/biopsy	34 (31)
No neck dissection (cN0)	53 (47)
Radiotherapy *	
Yes	21 (19)
No	91 (81)
**Site of recurrence**	
No recurrence	27 (24)
Local	53 (47)
Regional	13 (12)
Distant	19 (17)
**Year of treatment for recurrence**	
Before 2010	84 (75)
After 2010	6 (5)
Not recurrence	22 (20)
**Treatment for recurrence**	
No recurrence	27 (24)
Including surgery	60 (54)
Not including surgery	25 (22)

* photon-based in all cases

**Table 3:** Pathologic characteristics of the primary disease.

	N (%)
**Breslow**	
Median (first and third quartiles)	5.5 (3.4–9.0)
**Histologic type**	
Mucosal lentiginous	20 (18)
Nodular	20 (18)
Other	4 (3)
Not specified	68 (61)
**Pigmentation**	
Yes	14 (12.5)
No	98 (87.5)
**Cytologic features**	
Epithelioid	79 (71)
Other	17 (15)
Not specified	16 (14)
**Infiltration**	
None	26 (23)
Chorion	56 (50)
Bone	20 (18)
Other	10 (9)
**Ulceration**	
Absent	14 (13)
Present	73 (65)
Not specified	25 (22)
**Regression**	
Absent	74 (66)
Present	3 (3)
Not specified	35 (31)
**TILs**	
Absent	43 (38)
Brisk	12 (11)
Not brisk	28 (25)
Not specified	29 (26)
**Mitoses**	
Median (first and third quartiless)	5.0 (2.0–9.2)
**Lymphovascular invasion**	
Absent	68 (61)
Present	10 (9)
Not specified	34 (30)
**Vascular neogenesis**	
Absent	57 (51)
Present	22 (19)
Not specified	33 (30)
**Necrosis**	
Absent	56 (50)
Present	26 (23)
Not specified	30 (27)
**Perineural invasion**	
Absent	70 (63)
Present	5 (4)
Not specified	37 (33)
**Satellitosis**	
Absent	71 (63)
Present	1 (1)
Not specified	40 (36)
**Sclerosis**	
Absent	72 (64)
Present	9 (8)
Not specified	31 (28)
**Lateral surgical margins**	
No surgery	9 (8)
Negative	30 (27)
Positive	8 (7)
Not specified/not applicable	65 (58)
**Deep surgical margins**	
No surgery	9 (8)
Negative	30 (27)
Positive	8 (7)
Not specified/not applicable	65 (58)

**Table 4:** Results of the multivariable Cox models for overall (OS), disease-free survival (DFS), and post-relapse disease-free survival after first recurrence (prDFS).

	Overall Survival		Disease-Free Survival		Post-Relapse Disease-Free Survival	
	Hazard Ratio (95% CI)	*p*	Hazard Ratio (95% CI)	*p*-Value	Hazard Ratio (95% CI)	*p*
**Lymph node treatment within the curative setting**		<0.001		---		<0.001
Neck dissection vs. No neck dissection (cN0)	5.22 (2.39–11.40)		---		4.25 (2.21–8.18)	
**Primary tumor treatment**		0.077		---		---
Surgery vs. no surgery	0.24 (0.05–1.17)		---		---	
**Ulceration**		0.035		0.016		---
Present vs. absent	2.12 (1.05–4.26)		2.23 (1.16–4.28)		---	
**Sex**		0.051		0.058		0.079
Male vs. female	1.64 (1.00–2.71)		1.57 (0.98–2.49)		1.48 (0.96–2.28)	
**Radiotherapy**		0.249		---		---
Yes vs. no	1.48 (0.76–2.86)		---		---	

## Data Availability

The raw data supporting the conclusions of this article will be made available by the authors on request.

## Supplementary Materials

The following supporting information can be downloaded at: https://www.mdpi.com/article/10.3390/cancers18081304/s1, Figure S1. Survival curves of OS (panel A), DFS (panel B), and PFS from recurrence (panel C) stratified according to year of primary treatment (before 2010, after 2010 included). Figure S2. Survival curves of OS (panel A), DFS (panel B), and PFS from recurrence (panel C) stratified according to primary tumor site (nasal cavity, oral cavity, other). Figure S3. Survival curves of OS stratified according to sex (panel A: female, male), ulceration (panel B: ulceration, no ulceration), regional lymph node treatment (panel C: none, neck dissection, cN0), primary treatment including surgery (panel E: surgery, no surgery), radiotherapy (panel F: yes, no). Figure S4. Survival curves of DFS stratified according to sex (panel A: female, male), ulceration (panel B: ulceration, no ulceration), regional lymph node treatment (panel C: none, neck dissection, cN0), site of involved lymph nodes (panel D: cN0, cranial neck, medium neck), primary treatment including surgery (panel E: surgery, no surgery), radiotherapy (panel F: yes, no). Figure S5. Survival curves of prDFS stratified according to sex (panel A: female, male), ulceration (panel B: ulceration, no ulceration), regional lymph node treatment (panel C: none, neck dissection, cN0), site of involved lymph nodes (panel D: cN0, cranial neck, medium neck), primary treatment including surgery (panel E: surgery, no surgery), radiotherapy (panel F: yes, no).
