# Supplementary material for: Mucosal Melanoma of the Head and Neck: A 45-Year Experience of a Tertiary Cancer Center
Source: Cancers (Basel). 2026 Apr 20;18(8):1304. doi: 10.3390/cancers18081304 (PMC13115475; doi:10.3390/cancers18081304)
Supplement: Supplementary file 1 [file cancers-18-01304-s001.zip › cancers-4235218-supplementary.pdf]

Supplementary Figure captions

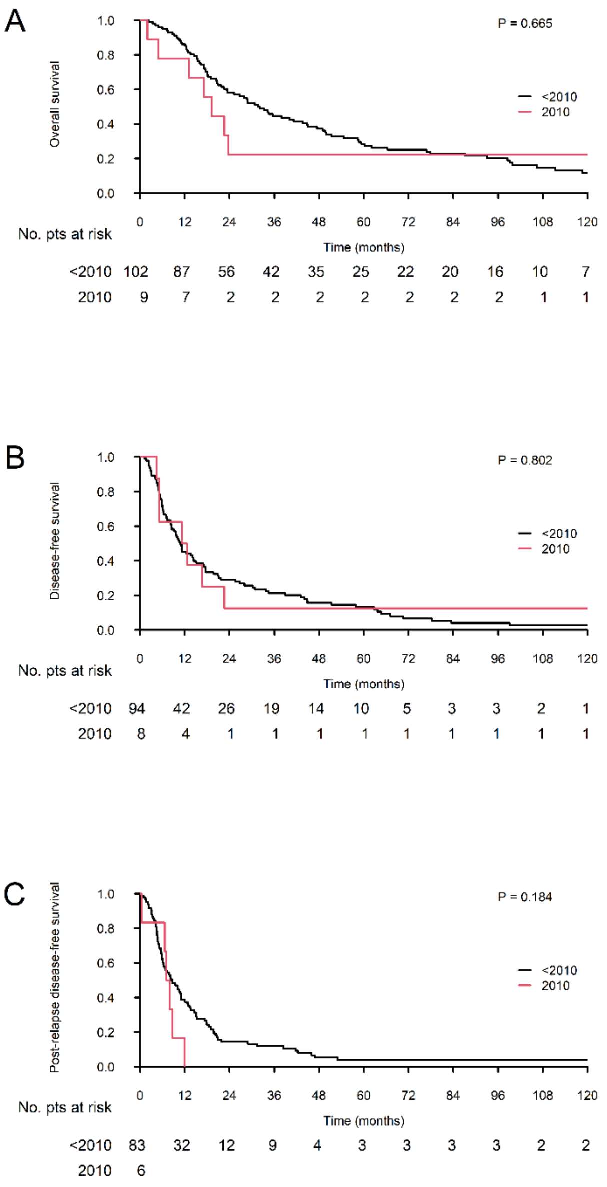

Supplementary Figure 1. Survival curves of OS (panel A), DFS (panel B), and PFS from recurrence (panel C) stratified according to year of primary treatment (before 2010, after 2010 included).

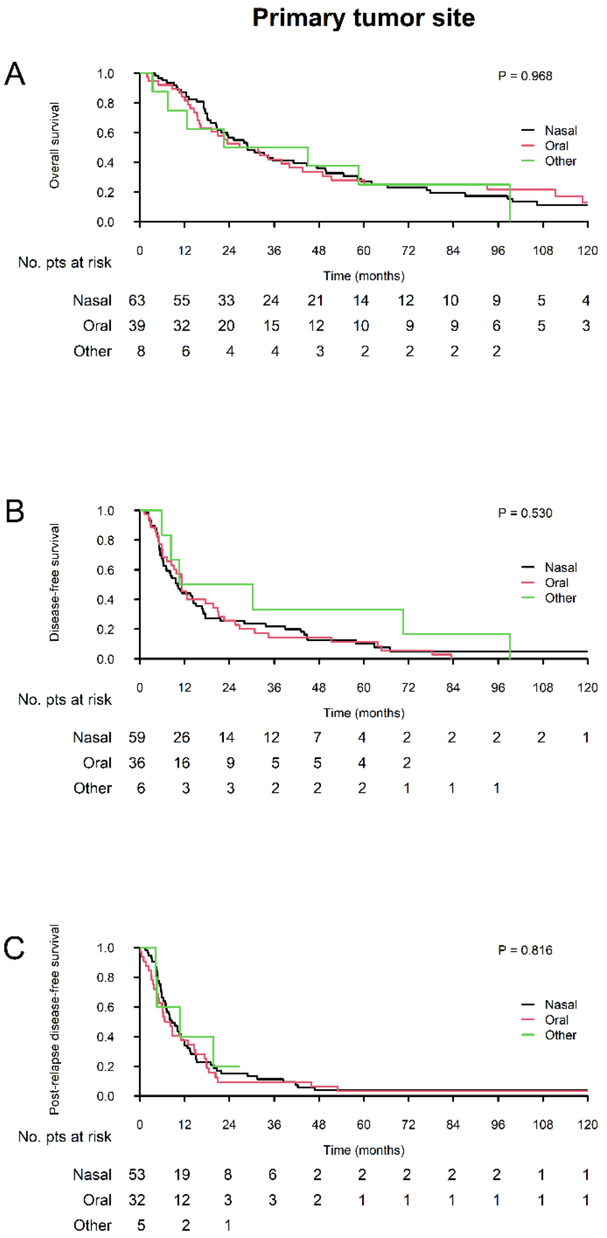

Supplementary Figure 2. Survival curves of OS (panel A), DFS (panel B), and PFS from recurrence (panel C) stratified according to primary tumor site (nasal cavity, oral cavity, other).

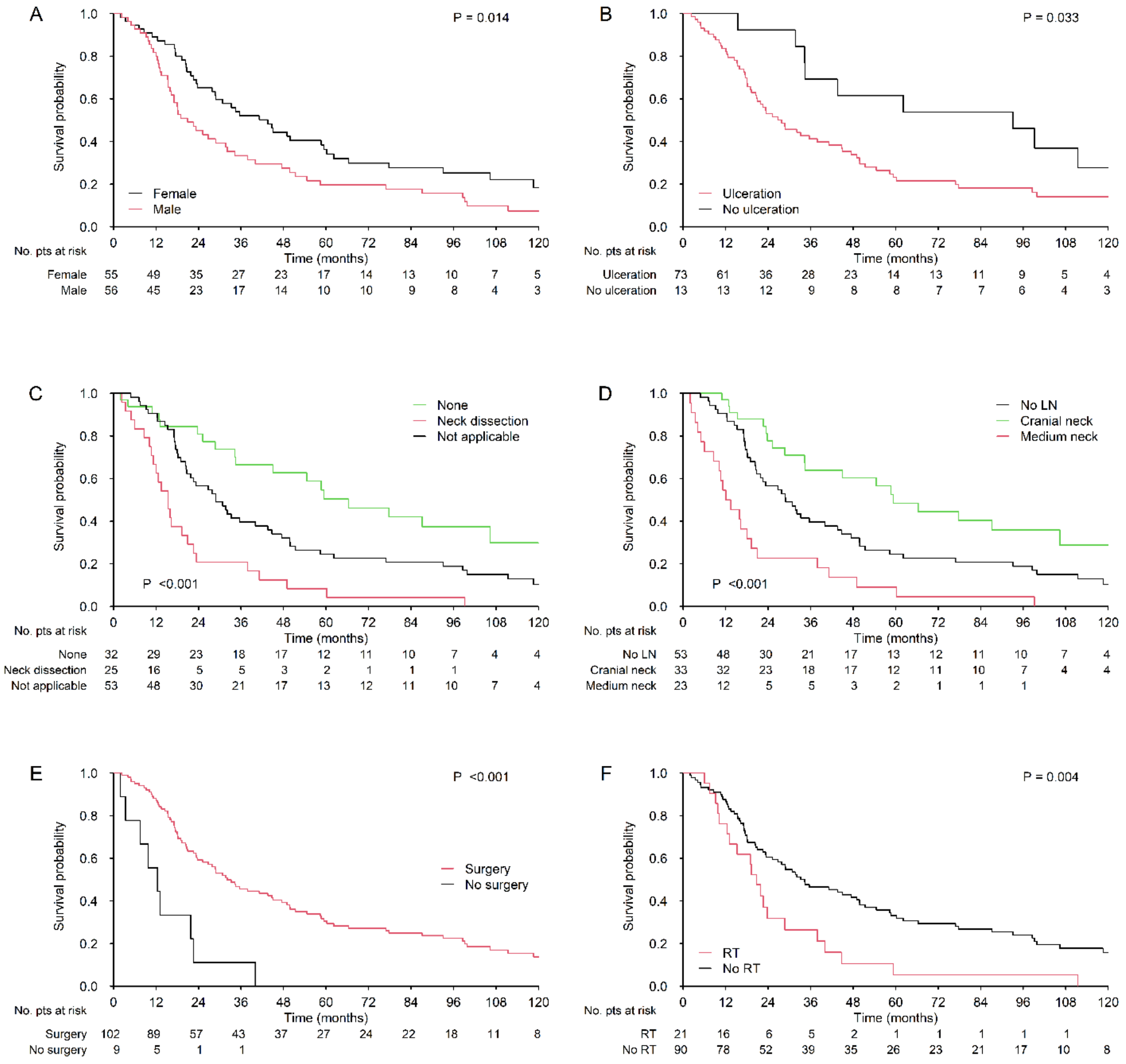

Supplementary Figure 3. Survival curves of OS stratified according to sex (panel A: female, male), ulceration (panel B: ulceration, no ulceration), regional lymph node treatment (panel C: none, neck dissection, cN0), primary treatment including surgery (panel E: surgery, no surgery), radiotherapy (panel F: yes, no).

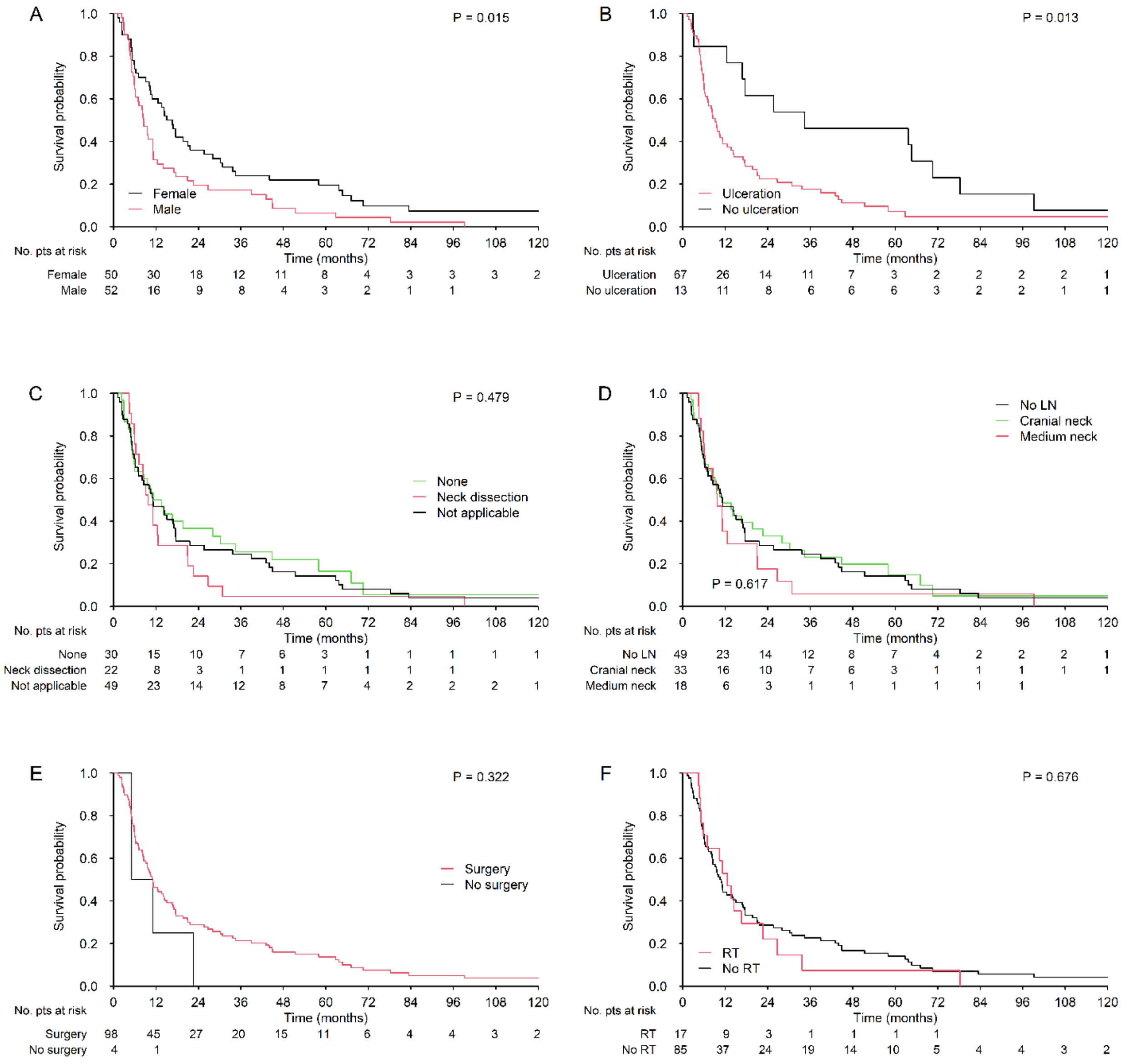

Supplementary Figure 4. Survival curves of DFS stratified according to sex (panel A: female, male), ulceration (panel B: ulceration, no ulceration), regional lymph node treatment (panel C: none, neck dissection, cN0), site of involved lymph nodes (panel D: cN0, cranial neck, medium neck), primary treatment including surgery (panel E: surgery, no surgery), radiotherapy (panel F: yes, no).

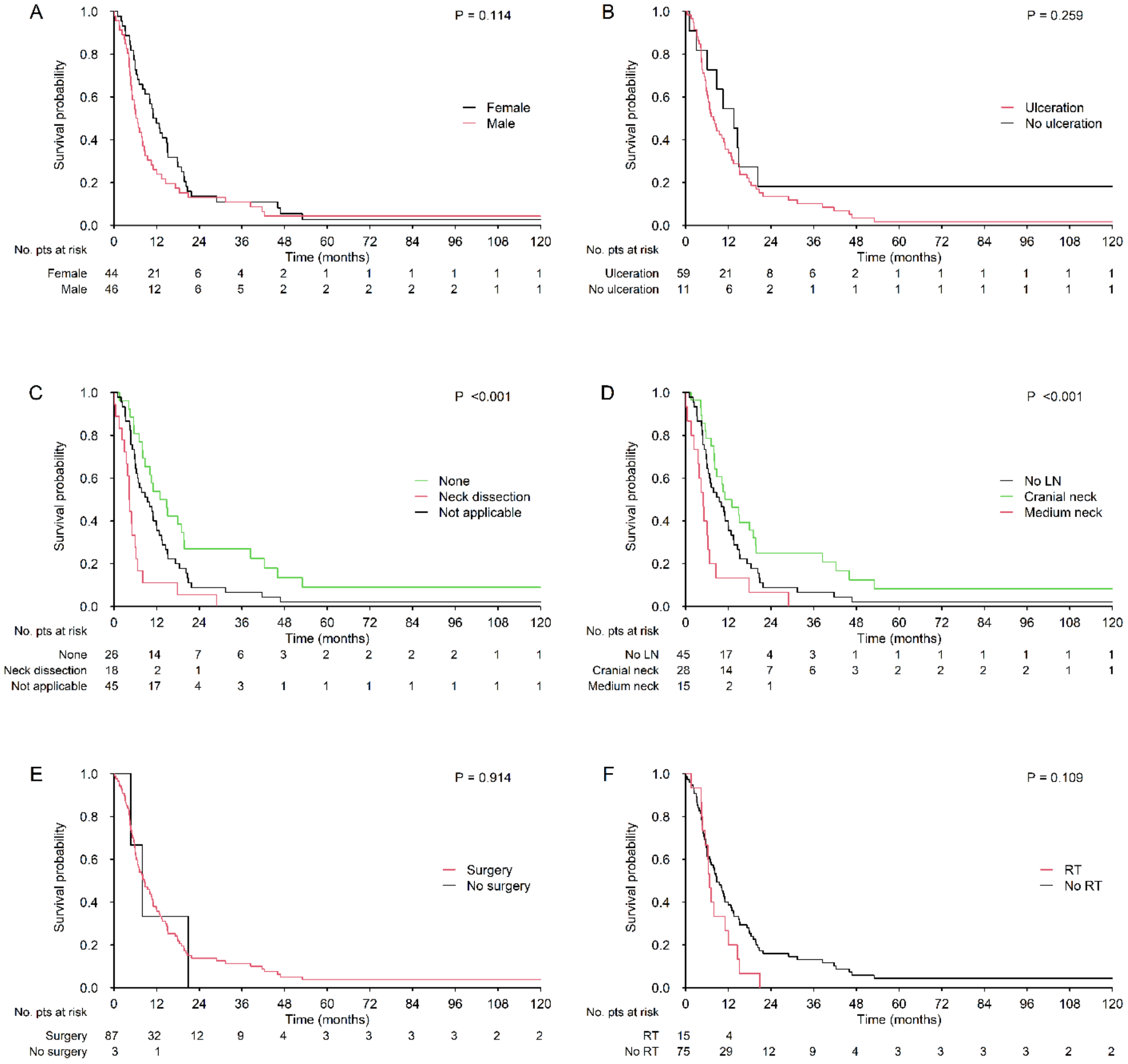

Supplementary Figure 5. Survival curves of prDFS stratified according to sex (panel A: female, male), ulceration (panel B: ulceration, no ulceration), regional lymph node treatment (panel C: none, neck dissection, cN0), site of involved lymph nodes (panel D: cN0, cranial neck, medium neck), primary treatment including surgery (panel E: surgery, no surgery), radiotherapy (panel F: yes, no).
